# Diagnostic performances of neutrophil to lymphocyte ratio and lymphocyte to monocyte ratio in acute ischemic stroke caused by cervicocranial arterial dissection

**DOI:** 10.1002/jcla.23515

**Published:** 2020-09-07

**Authors:** Yi Yang, Guangbi Sun, Shanshan Diao, Le Yang, Wanli Dong

**Affiliations:** ^1^ Departments of Neurology The First Affiliated Hospital of Soochow University Suzhou China; ^2^ School of Public Health Fujian Medical University Fuzhou China

**Keywords:** acute ischemic stroke, biomarkers, cervicocranial arterial dissection, diagnosis, lymphocyte to monocyte ratio, neutrophil to lymphocyte ratio

## Abstract

**Background:**

Inflammation plays an important role in the initiation and progression of cervicocranial arterial dissection (CCAD). New inflammatory indices derived from full cell blood count may be associated with increased risk of acute ischemic stroke (AIS) caused by CCAD. The goal of this study is to evaluate the diagnostic performances of neutrophil to lymphocyte ratio (NLR) and lymphocyte to monocyte ratio (LMR) in CCAD.

**Method:**

We retrospectively analyzed 52 patients with AIS caused by CCAD from emergency room (group I), 51 patients with CCAD from emergency room or clinic(group II) and 52 controls (group III), age and sex matched. Data were collected on the admission including NLR and LMR.

**Results:**

Neutrophil to lymphocyte ratio and LMR have significant differences among three groups, especially in group I vs both groups II and III (*P* < .001). There was a negative correlation between admission NLR and LMR. Low LMR level and high NLR level may be associated with severity of AIS caused by CCAD and significantly predict AIS in CCAD. The area under the curve of NLR and LMR were 0.77 and 0.71, respectively, on receiver operating characteristic curve analysis. The optimal cutoff values of NLR and LMR that best discriminated AIS were 2.35 (81% sensitivity and 63% specificity) and 3.67 (64% sensitivity and 77% specificity).

**Conclusions:**

Neutrophil to lymphocyte ratio neutrophil to lymphocyte ratio and LMR may contribute to the diagnostic evaluation and prompt immediate therapy in patients with CCAD.

## Graphical abstract

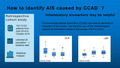

## 1 INTRODUCTION

As artery detachment, artery dissection refers to the vascular disease in which blood flow enters the artery wall and causes dissection of vascular wall.^1^ Cervicocranial arterial dissection (CCAD) can lead to stenosis or occlusion of the lumen has become one of the most frequent causes for ischemic stroke in the young.^1^, ^2^

Patients with CCAD are frequently complicated by coagulopathy and neurological complications, which consisted of permanent neurological dysfunction such as AIS and temporary neurological dysfunction such as headache, delirium, or a transient focal neurological deficit.^1^, ^3^ Magnetic resonance imaging (MRI) or computed tomography perfusion imaging is not always obtained for emergency cases although early diagnosis and timely treatment are essential for clinical practice. The application of carotid and cerebrovascular ultrasound increases the detection rate and the diagnostic accuracy of spontaneous CCAD. In general, we still lack a fast and effective way to identify AIS caused by CCAD and aid in risk stratification.

Inflammation plays an important role in the initiation and progression of cardio‐cerebrovascular diseases.^3^, ^4^ Studies show systemic inflammatory response will push forward an immense influence on the progression and outcome of AIS and aortic dissection(AD).^5^, ^6^, ^7^, ^8^ Blood parameters such as neutrophils, lymphocytes, and platelets can derange and influence their respective ratios, which were found to be predictors with diagnostic accuracy in AD, AIS, and other diseases.^5^, ^7^, ^9^, ^10^, ^11^ Among them, NLR and LMR are potential inflammatory biomarkers which have recently been reported as important predictors of overall survival in patients with tumor, intracerebral hemorrhage, or acute ischemic stroke.^12^, ^13^, ^14^, ^15^, ^16^ They are easily acquired blood markers through routine complete blood counts (CBC), posing little risk, or burden to the patient.

Dissection is an inflammation‐related disease. AIS cause by CCAD may have different inflammatory changes compared to AIS without CCAD. The aim of this study was to evaluate alterations of NLR and LMR levels in AIS caused by CCAD, investigating the diagnostic performance of these blood markers.

## 2 MATERIALS AND METHODS

### 2.1 Study population

A total of 103 patients (group I and group II) with cervicocranial arterial dissection in the First Affiliated Hospital of Soochow University from April 2014 to October 2019 were included into subsequent retrospectively analysis. CCAD was initially diagnosed by cervical and cerebral vascular ultrasound or computed tomography angiography (CTA) and was further confirmed by digital subtraction angiography (DSA) or high‐resolution magnetic resonance imaging (HR‐MRI). AIS was confirmed by MRI. The diagnosis was made by two senior imaging doctors. Exclusion criteria were as follows: (a) Patients had infection within 2 weeks before admission, cancer, chronic inflammation, hematological diseases, morbid obesity (BMI > 32), autoimmune diseases, or treatment with immunosuppressive agents; (b) patients occurred stroke within 6 months or the modified Rankin scale (mRS)>0 before the onset; and (c) patients cannot complete a blood count within 24 hours of admission. We compared them with 52 controls (group III), age, and sex matched. Data were collected from electronic patient records, and all patients gave informed consent.

### 2.2 Clinical information collection

We collected all study population's data, including demographic, medical histories, and clinical characteristics. Hypertension was determined by the previous use of antihypertensive medication, a systolic blood pressure ≥140 mm Hg, or a diastolic blood pressure ≥90 mm Hg. Diabetes was defined as previous use of hypoglycemic drugs, fasting blood glucose ≥7.0 mmol/L or postprandial blood glucose ≥11.1 mmol/L. Blood count analysis was carried out by autoanalyzer (Japan, Sysmex XS‐500i). The serum biochemical parameters were assayed by automatic biochemical analyzer (America, Siemens ADVIA 1800). Laboratory technicians were blind to the clinical characteristics or medical histories of the study population. The National Institutes of Health Stroke Scale (NIHSS) was used to assess the severity of stroke at admission and discharge.

### 2.3 Statistical analyses

Continuous variables were checked for the normal distribution assumption by Shapiro‐Wilk test. Then, they were analyzed as mean and standard deviation or the median and interquartile range properly. Differences among continuous variables were assessed by the Kruskal‐Wallis or variance analysis, post hoc analysis was performed with Bonferroni correction. Categorical variables were analyzed as frequency and percentage, and differences among these variables were assessed by the chi‐square test. Spearman rank correlation was used to evaluate the relationship among NLR, LMR, and stroke severity. Receiver operating curves (ROC) were analyzed to investigate the diagnostic performance of NLR and LMR. The level of significance for these descriptive comparisons was established at 0.05 for two‐sided hypothesis testing. Statistical analysis was performed in SPSS 25.0.

## 3 RESULT

The demographic and clinical characteristics of study population are showed in Table 1. There was no difference in age or gender among three groups. As expected, patients in groups I and II were more often with histories of smoking, diabetes, stroke or TIA and hyperlipidemia compared to controls. But there was no significant difference in history of hypertension or drinking among three groups.

**Table 1:** Demographic and clinical characteristics of patients with AIS by CCAD (group I), CCAD without AIS (group II), and controls (group III)

Characteristics	Group I	Group II	Group III	*P* value
Subjects, n	52	51	52	
Demographics				
Age in y, mean ± SD	46.06 ± 12.445	46.08 ± 11.88	48.98 ± 14.39	.42
Male, n (%)	32 (61.50)	22 (43.10)	33 (63.50)	.07
Smoking, n (%)	10 (19.20)	2 (3.90)	2 (3.80)	.01
Drinking, n (%)	5 (9.60)	3 (5.90)	2 (3.80)	.52
Medical history				
Hypertension, n (%)	24 (46.20)	20 (39.20)	15 (28.80)	.19
Diabetes, n (%)	5 (9.60)	10 (19.60)	2 (3.80)	.04
CHD, n (%)	0 (0.00)	1 (2.00)	0 (0.00)	.33
History of stroke or TIA, n (%)	10 (19.20)	3 (5.90)	0 (0.00)	<.001
Hyperlipidemia, n (%)	9 (17.30)	16 (31.40)	0 (0.00)	<.001
Clinical features				
Headache, n (%)	10 (19.20)	11 (21.60)	0 (0.00)	.002
Patients with vascular occlusion, n (%)	30 (57.70)	11.00 (21.60)	0.00 (0.0)	<.001
SBP in mm Hg, mean ± SD	129.7 ± 14.663	130.24 ± 19.86	126.35 ± 14.92	.43
DBP in mmHg, mean ± SD	79.90 ± 11.369	80.1 ± 9.92	79.10 ± 10.60	.88

Abbreviations: CHD, coronary heart disease; DBP,, diastolic blood pressure; IQR, interquartile range; SBP, systolic blood pressure; SD standard deviation.

Though systolic and diastolic blood pressure on admission were similar in all three groups, headache was more often in CCAD population than controls, and without difference between group I and group II of note.

**Figure 1:**
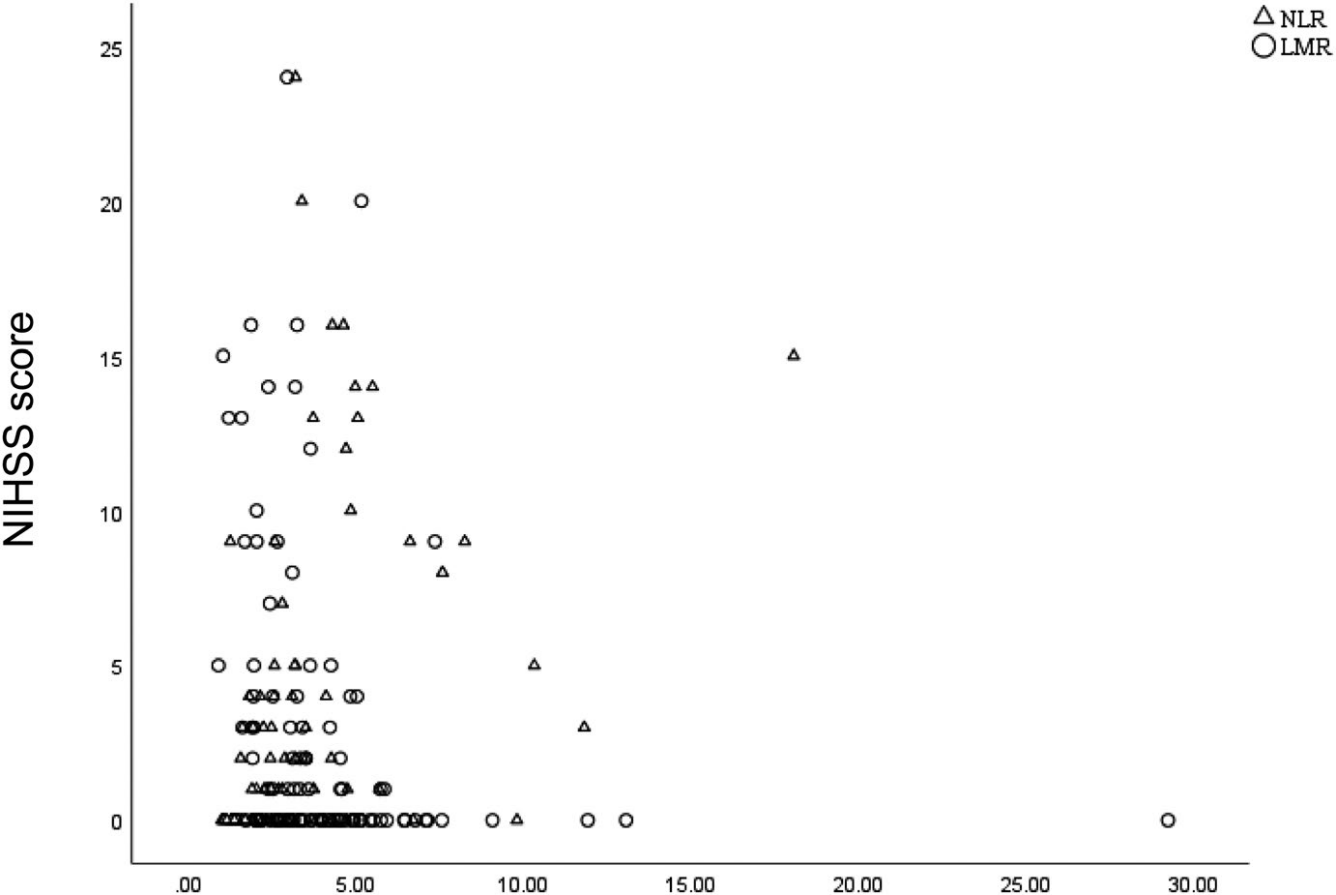
Correlation between NLR, LMR and NIHSS score. The NLR was positively correlated with the NIHSS (*r* = −.47, *P* < .001), and LMR level was negatively correlated with the NIHSS (*r* = −.41, *P* < .001), respectively. There was a negative correlation between admission NLR and LMR (*r* = −.51, *P* < .001). Abbreviations: NIHSS, National Institutes of Health Stroke Scale

The NLR level was positively correlated with the NIHSS score on admission (*r* = −.47, *P* < .001), and the LMR level was negatively correlated with the NIHSS score on admission (*r* = −.41, *P* < .001), respectively. And there was a negative correlation between admission NLR and LMR (*r* = −.51, *P* < .001) (Figure 1). High NLR and low LMR levels may be associated with severity of AIS caused by CCAD.

**Figure 2:**
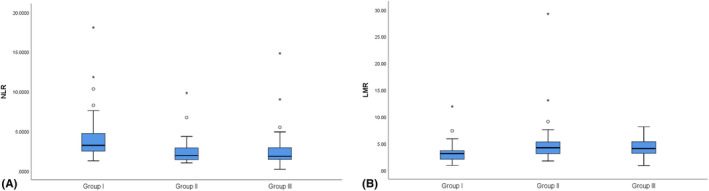
Box plots for NLR and LMR levels in three groups

**Figure 3:**
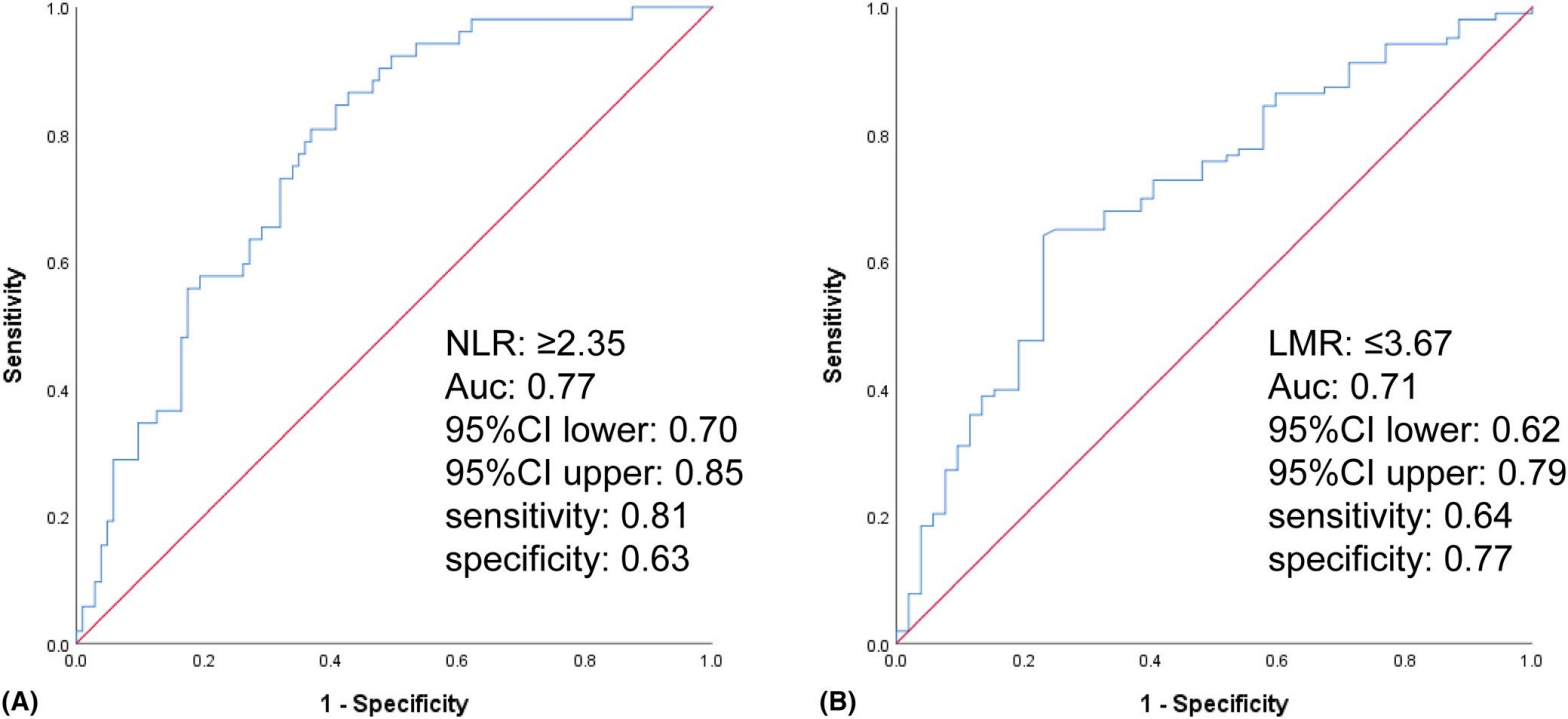
Receiver operating curve (ROC) showed predictive values of NLR and LMR for AIS in CCAD. (A, sensitivity = 0.81; specificity = 0.63; NLR = 2.35; AUC = 0.77; B, sensitivity = 0.64; specificity = 0.77; LMR = 3.67; AUC = 0.71)

We found that NLR is significantly higher in AIS by CCAD compared to both CCAD without AIS and controls (*P* < .001, Figure 2), with no significant difference between the last two groups (Table 1). The area under the curve was 0.77 (95%CI, 0.70‐0.84) on ROC curve analysis. The best cutoff value of NLR to predict AIS was 2.35 with 81% sensitivity and 63% specificity (Figure 3). For LMR, which is significantly lower in AIS by CCAD compared to both CCAD without AIS and controls (*P* < .001, Figure 2), with no difference between the last two groups (Table 1). The area under the curve was 0.71 (95%CI, 0.62‐0.79) on ROC curve analysis. The best cutoff value of LMR to predict AIS was 3.67 with 64% sensitivity and 77% specificity (Figure 3). NLR and LMR both show some certain predictive values for AIS caused by CCAD.

Likewise, in AIS by CCAD, WBC was higher as were the neutrophils and the monocyte (*P* < .05 for both subgroup). After adjustment, however, monocyte was higher in group I compared to group III (*P* = .004) and similar between I vs II and II vs III. Meanwhile, lymphocytes were highest in CCAD without AIS and have no significant difference between group I and group III (*P* < .05). After adjustment, it is similar between II and III. PLT was higher in group I compared to group III (*P* < .05) and similar between I vs II and II vs III (Table 1). But after adjustment, there was no longer statistical difference between our group (Table 2).

**Table 2:** Laboratory results in group I, group II, and group III

Laboratory results				
FGB in mmol/L, median (IQR)	5.17 (4.49, 5.88)	4.91 (4.47, 5.44)	4.81 (4.37, 5.38)	0.11
TC in mmol/L, median (IQR)	3.84 (3.30, 4.69)	3.98 (3.27, 4.83)	4.59 (4.13, 5.18)	0.002
TG in mmol/L, median (IQR)	1.27 (0.90, 1.68)	1.20 (0.83, 2.04)	1.20 (0.87, 1.73)	0.99
HDL in mmol/L, median (IQR)	1.05 (0.88, 1.24)	1.07 (0.91, 1.48)	1.07 (0.92, 1.32)	0.32
LDL in mmol/L, median (IQR)	2.26 (1.76, 2.85)	2.28 (1.80, 2.71)	2.63 (1.98, 3.12)	0.16
WBC in × 10^9^/L, median (IQR)	8.16 (6.84, 10.56)	6.29 (5.43, 7.92)	5.93 (4.50, 7.14)	<0.001
N in × 10^9^/L, median (IQR)	5.23 (4.02, 7.54)	3.66 (3.00, 5.12)	3.64 (2.62, 4.61)	<0.001
L in × 10^9^/L, median (IQR)	1.60 (1.27, 1.95)	1.93 (1.48, 2.26)	1.66 (1.35, 2.12)	0.02
M in × 10^9^/L, median (IQR)	0.57 (0.47, 0.67)	0.44 (0.34, 0.61)	0.43 (0.35, 0.50)	<0.001
NLR, median (IQR)	3.22 (2.49, 4.73)	1.95 (1.42, 2.92)	1.84 (1.44, 2.96)	<0.001
LMR, median (IQR)	3.13 (2.06, 3.66)	4.20 (3.08, 5.44)	4.05 (3.16, 5.36)	<0.001
CRP in mg/L, median (IQR)	2.58 (0.71, 6.46)	1.31 (0.57, 4.55)	0.84 (0.40, 1.63)	0.004
PLT in × 10^9^/L, median (IQR)	239.00 (195.00, 267.25)	219.00 (191.00, 262.00)	203.00 (168.75, 249.00)	0.09

Note

FGB fasting blood glucose (3.9‐6.1 in × mmol/L); TC total cholesterol (<5.2 in × mmol/L); TG triglyceride (<1.7 in × mmol/L); HDL high‐density lipoprotein cholesterol (≥1.0 in × mmol/L); LDL low‐density lipoprotein cholesterol (<3.4 in × mmol/L); WBC white blood cell (3.50‐9.50 in × 10^9^/L); N Neutrophil (1.80‐6.30 in × 10^9^/L); L Lymphocyte (1.10‐3.20 in × 10^9^/L); M Monocyte (0.10‐0.60 in × 10^9^/L); NLR neutrophil to lymphocyte ratio; LMR lymphocyte to monocyte ratio; CRP C‐reactive protein (0‐4 in × mg/L); PLT Platelet (125‐350 in × 10^9^/L).

## 4 DISCUSSION

We have shown, for the first time, NLR and LMR have significant differences in AIS by CCAD compared to CCAD without ischemic stroke and controls. These objective and fast biomarkers may improve our diagnostic accuracy for AIS caused by CCAD.

Inflammation plays a significant role in both pathogenesis of CCAD and stroke.^17^, ^18^, ^19^ Leucocytosis occurs as part of the acute inflammatory process in the vessel wall and carries prognostic significance.^20^, ^21^ NLR is a composite marker of absolute peripheral neutrophil and lymphocyte counts while LMR is a composite marker of absolute peripheral lymphocyte and monocytes counts. These cells comprise the total leukocyte count which play an important role in the inflammation and possibly in the pathogenesis of AIS and CCAD.^8^, ^17^, ^18^ Analyzing them apart may miss the interactions between these subtypes and their diagnostic performances in different medical conditions.

Elevated NLR which implies higher inflammatory burden signifies high neutrophil count due to active inflammation and low lymphocyte count correlating with defective response to the inflammatory process.^22^, ^23^, ^24^, ^25^ In previous studies, high neutrophil counts have been associated with adverse prognosis in AD and AIS, whereas high lymphocyte counts have been associated with protective effects in cerebrovascular patients.^6^, ^14^, ^26^ And among patients with AD and AIS, it has been shown that an increased NLR is a predictor of in‐hospital mortality and prognosis.^6^, ^27^ Though our data analysis, the NLR level was positively correlated with severity of stroke on admission. NLR was significantly higher in AIS by CCAD compared to both CCAD without AIS and controls, as were the leukocyte and the neutrophils. Our best cutoff value of NLR with a high degree of sensitivity but fairly low specificity was 2.35. Therefore, NLR is a novel parameter which may indicate inflammation and carry some diagnostic performances in CCAD. Meanwhile, lymphocytes were highest in CCAD without AIS. This may due to the regulatory function of some specific lymphocyte subsets in inflammation‐inducing neuroprotection,^26^ which need further exploration on modulating immune response to treat CCAD.

Similarly, LMR has been reported to be associated with adverse prognosis in multiple malignancies and cardio‐cerebrovascular disease.^12^, ^13^, ^28^, ^29^, ^30^ Low LMR signifies low lymphocyte count and high monocyte count. As another important immunoregulator different from lymphocyte, monocyte is involved into secondary injury following acute ischemic events.^31^ It is considered to differentiate into 3 major subtypes and classical monocyte, for instance, can promote vascular injury and neuronal death after AD and AIS by expressing pro‐inflammatory cytokines.^31^, ^32^ In previous retrospective analysis, higher proportion of monocyte after stroke was an independent predictor of 3‐month poor outcome.^12^ Our study showed, in CCAD patients, the LMR level was negatively correlated with the NIHSS score on admission and significantly lower in AIS by CCAD compared to both CCAD without AIS and controls. Inversely, marked monocytosis was observed in group I, which was in accordance with previous studies. Our best cutoff value of LMR with a high degree of specificity but fairly low sensitivity was 3.67. For patients with symptoms suggestive of acute neurological dysfunction, with, or without a history of CCAD, biomarkers exhibiting high specificity may be helpful to clinician to rule out AIS.

We compared AIS patients caused by CCAD with CCAD. Although pathogenesis and inflammatory indices may differ between the two subgroups, these CCAD patients share almost same clinical characteristics.^3^, ^33^, ^34^ In addition, this could be helpful in differentiating the cause of neurological complications. Our control group does not include normal volunteers but age and sex matched patients. Some of them have established risk factors for vascular disease; however, it may be more relevant in every day's clinical practice.

Our data should be interpreted with some caution due to limitations of the study. These include retrospective bias inherent to the study design and a small sample size. The association with race and metabolic diseases such as diabetes or nonalcoholic fatty liver disease have not been analyzed. And this study only analyzed the blood samples collected for the first time after admission. Further research need analysis the dynamic changes of this biomarkers during the course of CCAD.

## 5 CONCLUSION

This study suggests that NLR and LMR on admission as inflammatory biomarkers which might be useful in the diagnosis of AIS by CCAD. These reliable and easy‐to‐use predictors could contribute to clinical treatment strategy design in patients with CCAD. And further exploration on modulating immune response to treat CCAD are needed in the future.
